# Fermentation of Pea Protein Isolate by *Enterococcus faecalis* 07: A Strategy to Enhance Flavor and Functionality

**DOI:** 10.3390/foods14173065

**Published:** 2025-08-30

**Authors:** Zhunyao Zhu, Laijing Zhu, Yanli Wang, Ruixue Cao, Yifan Ren, Xiangzhong Zhao

**Affiliations:** School of Food Sciences and Engineering, Qilu University of Technology (Shandong Academy of Sciences), Jinan 250353, China; 18366260356@163.com (Z.Z.); 15725152458@163.com (L.Z.); 15106614358@163.com (Y.W.); 16355327129@163.com (R.C.); 15288886736@163.com (Y.R.)

**Keywords:** pea protein isolate, *Enterococcus faecalis*, fermentation, physicochemical properties, volatile compounds

## Abstract

Pea protein isolate (PPI) is a plant protein with high nutritional value, but its application in food is limited by an unpleasant beany flavor. This study aimed to investigate the feasibility of improving the flavor of PPI through fermentation with *Enterococcus faecalis* 07. PPI was subjected to fermentation by *E. faecalis* 07 for different durations (0 H, 24 H, 48 H, and 72 H). After fermentation, pH, viable cell counts, free amino acid contents, electronic tongue analysis, and volatile organic compounds were determined. The results showed that fermentation significantly reduced the bitterness of PPI and enhanced its umami intensity. A total of 64 volatile organic compounds were identified in the fermented samples, 42 more than in the unfermented sample. Quantitative analysis revealed that hexanal (grass-like odor) decreased by 92% after 72 h of fermentation, 1-octen-3-ol (mushroom-like odor) decreased from 6.94 mg/kg to 1.73 mg/kg, and trans-2-octenal decreased to 0.59 mg/kg; meanwhile, aromatic compounds such as esters and ketones were produced. Along with changes in the physicochemical properties, organic acids, and free amino acid composition of PPI, correlation analysis between electronic tongue data and volatile compounds further indicated that changes in volatile components simultaneously affected the perception of five taste attributes of PPI (bitterness, sourness, sweetness, saltiness, and umami). In conclusion, this study demonstrated the feasibility of fermenting PPI with *E. faecalis* 07, which effectively improved its sensory attributes and physicochemical properties to a certain extent.

## 1. Introduction

Beans (such as peas, lentils, lupins, and chickpeas), grains (such as wheat, corn, rice, barley, oats, and millet), and pseudograins (such as amaranth, buckwheat, and chia seeds) are common sources of plant protein [1]. Among them, compared with cereal protein, pea protein is more advantageous in terms of certain aspects [2],The amino-acid content is richer, the composition is more balanced, and the nutrition is more balanced [3]. However, the generation of undesirable volatile compounds such as hexanal and 1-octen-3-ol during the processing and storage of pea protein has also been recognized as a limiting factor for its consumer acceptance [4].

At present, there are three main methods for removing the smell of beans: physical methods, chemical methods, and biotechnological methods. The chemical method mainly inhibits the activity of lipoxygenase in PPI through treatment with chemical reagents, thereby achieving the purpose of removing the beany flavor of pea protein. Studies have demonstrated that extracting pea protein under different alkaline pH conditions resulted in a 57% reduction in hexanal content, as detected by SPME-GC/MS, while simultaneously leading to the formation of 1-octen-3-ol, an undesirable flavor compound [5]. However, chemical residues pose significant potential risks. Biotechnology includes genetic engineering, fermentation, and other approaches. For a long time, fermentation technology has been used to improve the sensory properties of plant-based proteins. Furthermore, studies have indicated that fermentation can also enhance the digestibility of plant proteins [6].

In recent years, lactic acid bacteria have been widely used as starters in fermented foods to enhance their nutritional content, sensory properties, and functionality, and also to improve consumers’ intestinal health [7]. The research conducted by Sun, Yang et al. [8] reported that Bifidobacterium animalis subsp. lactis 80 can effectively improve the volatile components such as aldehydes, furans, and esters in pea protein and simultaneously enhance the taste of pea protein. Pei et al. [9] reported that fermentation of pea flour with *Lactobacillus rhamnosus* L08 reduced the content of hexanal, a compound responsible for the beany flavor, by 80% after lactic-acid bacteria fermentation. Sophia Leffler et al. [10] employed six different lactic-acid bacteria strains to ferment PPI. The results indicated that with increasing fermentation time, the bitterness and saltiness of PPI were alleviated, and its functionality was also improved. However, fermentation with *L. fermentum* resulted in a fecal odor. Chung. Eun, Su Cheol et al. [11], conducted a study on the fermentation of soy milk by Lactobacillus plantarum. They not only improved the sensory properties of the soy milk but also enhanced its nutritional quality, achieving some favorable results. Another study has shown that cheese fermented with Enterococcus faecalis exhibited significant improvements in both texture and sensory attributes starting from day 15 [12]. Furthermore, it has been reported that fermentation of soy milk with *Enterococcus faecium* 29309 led to a reduction of acetaldehyde content to as low as 10 mg/L by day 7, accompanied by the production of 2,3-butanediol, a compound contributing a buttery flavor. This fermentation process enhanced the texture and rheological characteristics of the soy milk [13]. It can also promote the intestinal health of consumers [14].

Although previous studies have predominantly focused on fermentation using lactic-acid bacteria, the fermentation of PPI with Enterococcus faecalis has scarcely been investigated. Therefore, the present study aimed to investigate the feasibility of fermenting pea protein isolate (PPI) with *Enterococcus faecalis* 07, with the objectives of reducing undesirable volatile compounds associated with beany, mushroom-like, or grassy flavors, such as hexanal and 1-octen-3-ol, and evaluating whether *Enterococcus faecalis* 07 can serve as an effective starter culture to improve the content of free amino acids, flavor-enhancing nucleotides, and the functional properties of PPI. This study provides guidance for the application of *Enterococcus faecalis* 07 in plant-based foods and offers a reference for the future utilization of pea protein in food processing.

## 2. Materials and Methods

### 2.1. Materials and Strains

Pea protein isolate (PPI) was obtained from YuWang (Dezhou, China). *Enterococcus faecalis* 07 was preserved in our laboratory. Soybean oil was purchased from Jiajia Yue (Jinan, China). MRS broth medium and agar were both provided by Beijing Aobo Xing Biotechnology Co., Ltd. (Beijing, China). Lactic acid standard was purchased from Hefei Boheme Biotechnology Enterprise (Hefei, China), and organic acids such as malic acid and tartaric acid were purchased from Maclyn. 5-Sulfosalicylic acid dihydrate was from Maclyn. 5′-AMP, 5′-GMP, and 5′-CMP nucleotide standards were purchased from Yuanye Biotechnology Company (Shanghai, China). The bovine serum albumin was purchased from Solarbio Co., Ltd. (Beijing, China). All other analytical reagents were purchased from Sigma-Aldrich Co., Ltd. (St. Louis, MO, USA).

### 2.2. The Fermentation of Pea Protein

Ten grams of pea protein isolate (PPI) were thoroughly mixed with 150 mL of deionized water and transferred into a 250 mL conical flask. The mixture was sterilized at 121 °C for 15 min to remove endogenous microorganisms. Under sterile conditions, the strain (3%, *v*/*v*) was added to the pea-protein suspension, which was then placed in a constant temperature incubator (SHX 150 III, Shanghai, China) for fermentation for 24, 48, and 72 h. All samples were subjected to viable cell counts, pH measurement, and organic acid determination before freeze-drying.

### 2.3. Microbial Analysis

The number of bacteria was determined by taking 0.5 mL of the pea protein fermentation broth. For each fermentation sample, three biological replicates (i.e., samples from three independent fermentation experiments) were prepared. Each biological replicate was diluted with sterile water at a ratio of 1:10 and then plated on MRS agar medium in three technical replicate plates. The plates were incubated at 37 °C under aerobic conditions for 48 h, and colonies were counted. Viable cell counts are expressed as Log CFU mL^−1^.

The pH of each sample was measured by taking 20 mL of the fermented pea protein solution. Each sample was analyzed in three biological replicates, and for each biological replicate, three technical replicate measurements were performed using a pH meter (PB-10). All measurements were temperature-compensated.

### 2.4. Functional Properties of Proteins

#### 2.4.1. Solubility

The experiment was conducted based on the method of Kitts et al. [15] with modifications. A total of 0.5 g of freeze-dried samples were mixed with 30 mL of distilled water and were stirred at room temperature for 1 h. The mixture was subsequently centrifuged at 7000 rpm for 15 min (TGL-15B). Protein concentration in the resulting supernatant was determined using the Bradford assay to assess protein solubility. Bovine serum albumin (BSA) was used to construct a standard curve. Protein solubility was expressed as the percentage of protein present in the supernatant relative to the total protein content.

#### 2.4.2. Water Retention Capacity/Oil Retention Capacity

The experiment was conducted according to the method described by Luo, Fan et al. [16] with slight modifications. Take 0.5 g of PPI in a pre-weighed centrifuge tube, add 10 mL of distilled water and soybean oil, vortex for 15 min, then let the mixture stand for 5 min, centrifuge at 8000 r/min for 15 min (TGL-15B), remove the supernatant, and weigh the residue.

### 2.5. Determination of Organic Acid Content

The detection of organic acids was based on the method of Feng, Liu et al. [17] and was modified accordingly. One milliliter of fermentation broth was mixed with 1 mL of deionized water (1:1, *v*/*v*). The mixture was centrifuged at 4 °C and 6000 rpm for 15 min (TGL-15B) to obtain the supernatant. The supernatant was then filtered through a 0.22 μm membrane and aliquoted into HPLC sample vials. High-performance liquid chromatography (HPLC, Shimadzu, Kyoto, Japan) was performed using an Agilent C18 column (4.6 mm × 250 mm, 5 μm) with a detection wavelength of 210 nm and a column temperature of 40 °C. The mobile phases consisted of 0.2% phosphoric acid solution (A) and methanol (B, chromatography grade), with a flow rate of 0.5 mL/min.

### 2.6. Determination of Free Amino Acids

Based on the method described by Xiong, Xu et al. [18], with minor modifications, the free amino acids were determined. In brief, 1.0 g of the sample was placed in a 10 mL centrifuge tube, and 9 mL of 5-sulfosalicylic acid (7%, *w*/*v*) was added and then vortexed for 10 min before being left to stand for 15 min. The tube was then centrifuged (7000 r/min, 15 min), and the analysis was conducted using an automatic amino-acid analyzer.

### 2.7. 5 Nucleotide Detection

The method proposed by Xue, Wang et al. [19] was modified as follows: 2 g PPI were mixed with 15 mL of 5% perchloric acid and homogenized for 3 min. The mixture was allowed to stand at room temperature for 20 min, followed by centrifugation at 9000 rpm for 15 min at 4 °C (TGL-15B). The supernatant was collected, and the extraction was repeated twice with 15 mL of 5% perchloric acid. All supernatants were combined, and the pH was adjusted to 6.5–7.0 using KOH. The final volume was then brought to 100 mL with deionized water. The solution after volume stabilization was filtered through a 0.22-μm filter and was analyzed by HPLC. The mobile phases were 0.05 mol/L KH2PO4 and methanol, respectively, the column of chromatography was Agilent C18 (4.6 mm × 250 mm, 5 μm), the flow rate was 0.9 mL/min, the column temperature was 35 °C, and the detection wavelength was 254 nm.

### 2.8. Determination of Volatile Organic Compounds

This was conducted according to the method described by Qiang, Zhao et al. Qiang, Zhao et al. [20] with some modifications. After mixing 1.5 g of pea protein isolate sample with 4 mL of distilled water in a headspace vial, 2 μL of 2,6-dimethylpyridine (Maclyn, Shanghai, China) as an internal standard was added and the mixture was stirred. The sample was equilibrated at 60 °C for 15 min, and then 45 min of extraction was performed using an aged SPME fiber extraction needle (250 °C, 30 min) (Merck Supelco, Shanghai, China) at 60 °C. The temperature program for the coupled quadrupole time-of-flight mass spectrometer (Thermo Fisher, TSQ 8000 Evo, Guangzhou, China) was as follows: the column was maintained at 40 °C for 6 min, ramped at 4 °C/min to 80 °C, then at 2 °C/min to 86 °C and held for 3 min. Subsequently, the temperature was increased at 4 °C/min to 120 °C and held for 2 min, followed by a ramp of 10 °C/min to 250 °C and it was maintained for 5 min. Helium was used as the carrier gas at a flow rate of 1 mL/min. Mass-spectrometry conditions were set as follows: ion source temperature—280 °C, electron energy—70 eV, scan mass range—35–1050 m/z, ion transfer line temperature—280 °C, and split ratio—2:1.

### 2.9. E-Tongue Analysis

Following the method in [21] and making modifications, the test was conducted using an electronic tongue instrument (INSENT SA402B, Atsugi, Japan). A total of 0.2 g of the sample was weighed and added to 100 mL of distilled water. After shaking for 10 min, it was filtered and then sent for testing. This test utilized five taste-detection sensors, namely, AAE (umami), CT0 (saltiness), GL1 (sweetness), CA0 (acidity), and COO (bitterness).

### 2.10. Sensory Evaluation

A sensory panel consisting of eight members (four males and four females, aged 23–35) from the School of Food Science and Engineering at Qilu University of Technology participated in the evaluation of aroma characteristics using Quantitative Descriptive Analysis (QDA). Informed consent was obtained from each panelist prior to the sensory evaluation, and all participants were required to complete a specific training program aimed at improving their proficiency in identifying and describing aroma attributes.

Based on a modified version of a previously established method [22], six odor attributes (aromatic, beany, fatty, caramel-like, sour, and meaty) were evaluated. All test samples were randomly assigned three-digit codes and were presented to the panelists in triplicate for scoring.

The ethical approval for sensory evaluation was granted by the Ethics Committee of the School of Food Science and Engineering, Qilu University of Technology, and covered all participants.

### 2.11. Data Analysis

All measurements were made in triplicate and the result values were expressed as the mean ± SD. Statistical analysis was performed using one-way ANOVA. All statistical analyses were conducted using IBM SPSS v26. The significance level was set at *p* < 0.05. To analyze the flavor differences, SIMCA 14.1 software was used for analysis.

## 3. Results and Discussion

### 3.1. Microbial, pH, and Organic Acid Analysis

**Figure 1:**
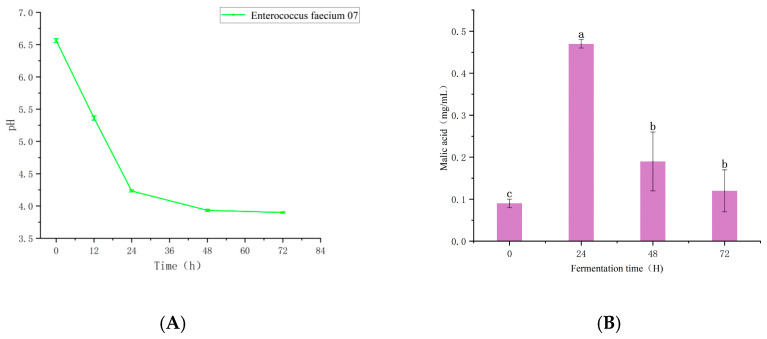

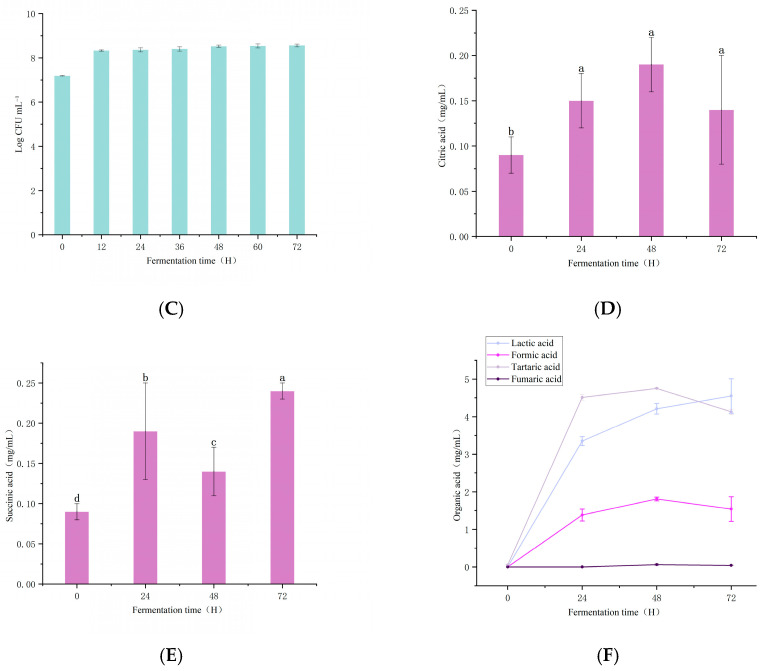
*Enterococcus faecalis* 07 The changes in colony count and pH (**A**), the changes in malic-acid content during fermentation (**B**). Changes in microbial counts over fermentation time (**C**).The changes in citric-acid content during fermentation (**D**). The changes in succinic-acid content during fermentation (**E**). The changes in lactic-acid, formic-acid, tartaric-acid, fumaric-acid content during fermentation (**F**). Different letters within the same column represent significant differences among samples (*p* < 0.05).

As shown in Figure 1C, the viable cell counts in the fermentation broth increased progressively over the course of fermentation. Specifically, during the initial 72 h, the viable cell count of *Enterococcus faecalis* 07 increased significantly from 7.19 Log CFU mL^−1^ to 8.56 Log CFU mL^−1^ (*p* < 0.05). These results indicated that *E. faecalis* 07 was able to ferment using pea protein as the substrate.

Due to the production of organic acids such as tartaric acid, lactic acid, and malic acid during fermentation, the pH value continuously decreased during the fermentation process. After 24 h of fermentation, the pH value of the pea protein fermentation liquid significantly decreased (*p* < 0.05) and then decreased slowly. This might be because the nutrients were consumed during the fermentation process [23].

The changes of organic acid contents during the fermentation process of pea protein are shown in Table 1. Following fermentation, seven types of organic acids were detected in the pea protein, including malic acid, lactic acid, citric acid, succinic acid, and fumaric acid, etc. At 48 h of fermentation, the tartaric-acid content reached the highest level (4.7 mg/mL), showing a significant difference compared with the unfermented PPI (*p* < 0.05), followed by lactic acid and formic acid. During the fermentation period of 72 h, the lactic-acid content increased continuously, rising from 0.07 mg/mL at the beginning of fermentation to 4.60 mg/mL at the end of fermentation, with a statistically significant difference (*p* < 0.05). This accumulation is likely associated with the malate–lactic acid fermentation pathway [24], This approach is particularly common in the wine-making process. It also explains the trend of changes in malic acid. Moreover, malic acid–lactic acid fermentation not only inhibits some spoilage microorganisms but also produces flavor substances such as esters and alcohols, which may help improve the quality of fermented pea-protein products [25]. Furthermore, previous studies have shown that lactic acid not only enhances food flavor but also plays a role in alleviating obesity- and diabetes-related symptoms [26]. Formic acid shows an initial increase during the fermentation process, and then takes a turn after 48 h of fermentation, presenting a downward trend. It decreases from 1.82 mg/mL at 48 h to 1.54 mg/mL. This might be due to the formation of formic acid heptanoate as a result of the reaction of formic acid during the fermentation process. In contrast, the concentrations of fumaric acid remained relatively stable throughout the process. Notably, succinic acid, known for its umami-enhancing and viscosity-improving properties [27], increased from 0.09 mg/mL to a peak of 0.24 mg/mL after 72 h of fermentation. This increase may contribute to masking or improving the off-flavor characteristics commonly associated with pea protein.

### 3.2. Protein Properties

#### 3.2.1. Solubility

The research has shown that, compared with the unfermented PPI, the solubility of the fermented PPI has significantly decreased (*p* < 0.05), which is consistent with the experimental results of Yuan Yuan et al. [15]. After 24 h fermentation, the solubility of PPI reached its lowest point, dropping from the initial 8.6% to 3.2%. However, there was no significant change in the solubility of PPI among different fermentation time periods (*p* > 0.05). Experimental evidence shows that the lowest solubility of PPI occurs at the isoelectric point [28]. According to the analysis of pH changes during fermentation, the pH gradually deviated from the isoelectric point as the fermentation time increased. In addition, the pH reduction induced by fermentation caused PPI to adopt a looser molecular structure under low-pH conditions, with more hydrophobic groups exposed on its surface [29], thereby resulting in a decrease in solubility.

#### 3.2.2. Water-Retention Capacity/Oil-Retention Capacity

**Figure 2:**
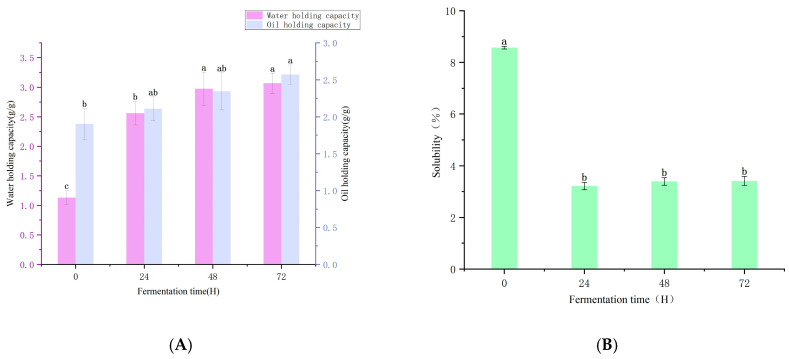
The water-retaining and oil-retaining properties of PPI change during fermentation (**A**). The solubility of PPI changes during fermentation (**B**). Different letters within the same column represent significant differences among samples (*p* < 0.05).

Water-holding capacity (WHC) is an indicator of protein–water interaction and is helpful for maintaining water in protein foods. According to the results shown in Figure 2A, the WHC and oil-holding capacities of PPI after fermentation were significantly higher than those of untreated PPI (*p* < 0.05). Research shows that after 24 h of fermentation, the water-holding capacity of PPI increased from the initial 1.13 (g/g) to 2.56 (g/g). Subsequently, as time passed, the water-holding capacity continued to increase until it reached 3.07 (g/g) at 72 h. However, from Figure 2B, it can be seen that the solubility of PPI was lower than that of the unfermented PPI, This phenomenon whereby the protein’s water-holding capacity does not conform to the trend of protein solubility change is consistent with the experimental results of Zhu and Sun et al. [30], which indicate that protein solubility has no direct relationship with water-holding capacity (WHC), and high protein solubility does not necessarily mean high water-holding capacity. This might be attributed to a lower number of α-helical structures [31]. Simultaneously, fermentation may have induced structural denaturation of PPI, leading to the exposure of additional hydrophilic groups, thereby enhancing its capacity to bind water molecules [32]. These factors led to the situation that the solubility of PPI after fermentation remained relatively high even when it was lower than that of the untreated PPI and still maintained a high WHC.

The ability of protein food to retain oil can be reflected by the oil-holding capacity (OHC). The OHC of all the fermented samples was higher than that of the untreated PPI, which was consistent with the research results of this trend [33]. With the variation of fermentation time, the oil-retention capacity at 72HPPI increased to 2.57 (g/g), which was significantly higher than 1.93 (g/g) without treatment (*p* < 0.05). This might be because the solubility of PPI decreased significantly within 72H of fermentation, increasing the hydrophobicity of proteins, thereby promoting the binding between proteins and oil [34]. In conclusion, after fermentation, the water-holding capacity and oil-holding capacity of PPI significantly increased compared to before fermentation. This is of great help for the development of protein-based foods.

### 3.3. Nucleotide Analysis

**Figure 3:**
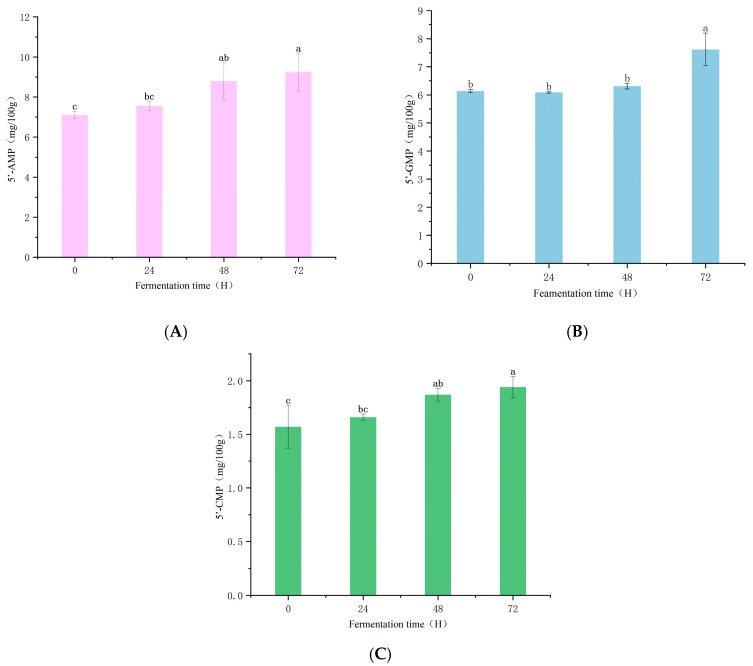
Changes of 5′-AMP during fermentation (**A**). Changes of 5′-GMP during fermentation (**B**). Changes of 5′-CMP during fermentation (**C**). Different letters within the same column represent significant differences among samples (*p* < 0.05).

Umami taste can also be contributed by 5′-nucleotides. In this study, the changes in 5′-AMP (adenosine monophosphate), 5′-GMP (guanosine monophosphate), and 5′-CMP (cytidine monophosphate) in pea protein were examined. As shown in Table 2, compared with the pre-fermentation state, the content of GMP did not exhibit a significant increase in PPI after 24 h of fermentation (*p* > 0.05). With the extension of fermentation time, the peak value was found at 72 h, which was 7.62 (mg/100 g). It has been reported that the umami and savoriness are positively correlated with the concentration of GMP [35]. Furthermore, the presence of glutamate will significantly enhance the umami flavor of GMP [36] and has a beneficial effect on the taste of PPI. 5′-AMP gives food a fresh flavor, thereby influencing the taste of food [37]. Meanwhile, 5′-AMP can also provide sweetness and inhibit the bitter taste of food. According to the table, compared with 5′-GMP and 5′-CMP, the content of 5′-AMP is the highest, followed by 5’-GMP and 5’-CMP. In addition, the concentration of 5’-AMP in untreated PPI is 7.10 (mg/100 g), and after fermentation treatment, the concentration of 5’-AMP in PPI continuously increases. After 24 h and 48 h of fermentation, the concentration of 5’-AMP increases to 7.55 (mg/100 g) and 8.81 (mg/100 g), respectively, and the maximum value of 9.24 (mg/100 g) is observed after 72 h of fermentation. It may be that IMP is degraded through the de novo nucleotide synthesis pathway, thereby generating 5′-AMP [38]. The content of 5′-CMP is the lowest, but as shown in the Figure 3, within 72 h of fermentation, 5′-CMP is also continuously increasing. After 72 h of fermentation, it significantly increases from the initial 1.57 (mg/100 g) to 1.94 mg/100 g (*p* < 0.05). As indicated in Chen, Fang et al. [39], in the fermentation process of shiitake mushrooms, the concentration of 5′-CMP is the highest, which may be the main contributor to the flavor of shiitake mushrooms.

### 3.4. Analysis of Free Amino Acids

**Figure 4:**
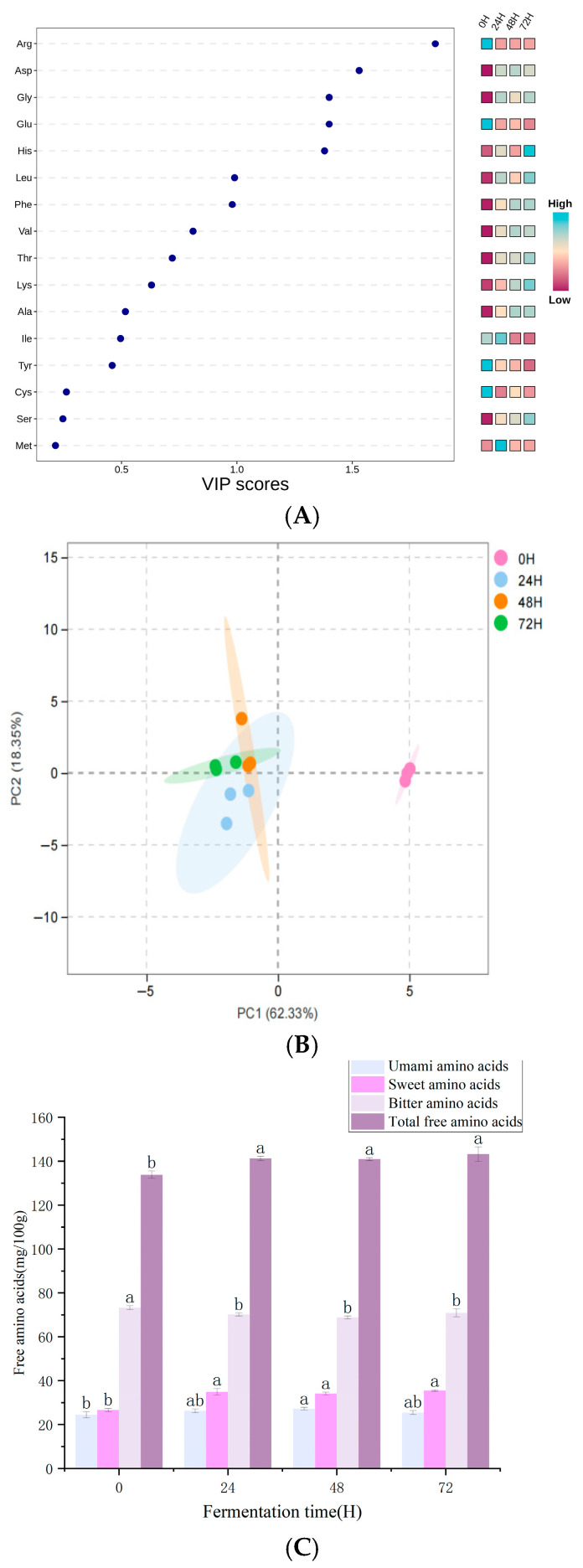
Comparison of free-amino-acid profiles of different fermented PPI samples. VIP score plots from PLS-DA (**A**). PCA plot (**B**). PC 1 and PC 2 represent principal component 1 and principal component 2, respectively. Total amounts of bitter amino acids, sweet amino acids and umami amino acids, and free amino acids change with fermentation time (**C**). 0H, 24H, 48H, and 72H represent the fermentation time of PPI, respectively. Different letters within the same column represent significant differences among samples (*p* < 0.05).

In this study, the contents of free amino acids in both unfermented and fermented PPI were determined. Principal component analysis (PCA) revealed a marked difference in the free-amino-acid profiles between PPI fermented by cocci and unfermented PPI. Furthermore, as shown in Figure 4C, the total free-amino-acid content of PPI was significantly increased after fermentation compared with that of unfermented PPI (*p* < 0.05). This phenomenon can be attributed to the hydrolysis of proteins by extracellular proteases secreted by microorganisms into small peptides, which are subsequently degraded by extracellular peptidases into various free amino acids [13,40].

Partial least squares discriminant analysis (PLS-DA) of the variable importance in projection (VIP) scores indicated that arginine (Arg), aspartic acid (Asp), glutamic acid (Glu), and glycine (Gly) were the free amino acids most affected by fermentation (VIP ≥ 1) (Figure 4A). A similar phenomenon was also observed by Li, Guo et al. [13] during the fermentation of soymilk with *Enterococcus faecium* CGMCC29309. Among these, Arg was the most strongly affected, with its content decreasing by more than twofold during fermentation. This reduction may be attributed to the arginine decarboxylase pathway of *Enterococcus faecium* [41]. Moreover, Arg is a well-recognized bitter amino acid, and its reduction contributes to the mitigation of the bitter taste of PPI [42]. Interestingly, compared with other studies on fermentation by *Enterococcus faecium* [13,42], Glu did not accumulate in our study but instead showed a significant decrease with prolonged fermentation time (*p* < 0.05). Previous studies [43,44], have reported that *Enterococcus faecium*, as a type of lactic-acid bacterium, can catalyze Glu through glutamate decarboxylase, which may account for the observed reduction in Glu. In addition, Table 3 clearly shows that the content of Gly peaked at 72 h, reaching a significantly higher level than that of the unfermented sample. A similar result was also reported by Qiu, Wu et al. [45], which was attributed to the presence of glycine hydroxymethyltransferase in microorganisms, enabling the conversion of serine to glycine. Furthermore, in this study, the levels of Cys, Val, and Met initially increased and then decreased; however, their final concentrations were still significantly higher than those in unfermented PPI, which may be related to the relatively short fermentation period [46].

Free amino acids can be classified into sweet, umami, and bitter ones based on their flavor. Among them, glutamic acid (Glu) and aspartic acid (Asp) are related to umami, while serine (Ser), alanine (Ala), threonine (Thr), and glycine (Gly) are identified as related to sweetness. Isoleucine (Ile), valine (Val), leucine (Leu), arginine (Arg), phenylalanine (Phe), histidine (His), methionine (Met), and tyrosine (Tyr) are associated with bitterness [47]. According to Figure 4C, among the flavor amino acids, the content of bitter amino acids is the highest. During the fermentation process, the content of bitter amino acids shows a trend of rising first and then falling, decreasing from 73.32 (mg/100 g) before fermentation to 70.93 (mg/100 g) at the end. Sweet and umami amino acids significantly increased overall after fermentation, increasing from 26.7 (mg/100 g) and 24.51 (mg/100 g), respectively, to 35.57 (mg/100 g) and 25.51 (mg/100 g).

### 3.5. Volatile Organic-Compounds Analysis

To gain deeper insights into the variations of volatile components during the fermentation of pea protein, solid-phase microextraction coupled with gas chromatography–mass spectrometry (SPME-GC/MS) was employed to systematically analyze and identify the aroma compounds and their relative contents in both unfermented and fermented pea-protein samples. These volatile compounds were broadly classified into seven categories: aldehydes, ketones, esters, acids, alcohols, pyrazines, and others. Venn-diagram analysis revealed that 17 volatile components were common to both unfermented and fermented pea-protein samples. Notably, the unfermented pea protein contained a higher proportion of aldehydes and ketones, which are recognized as the primary contributors to the characteristic off-flavors of PPI [48].

**Figure 5:**
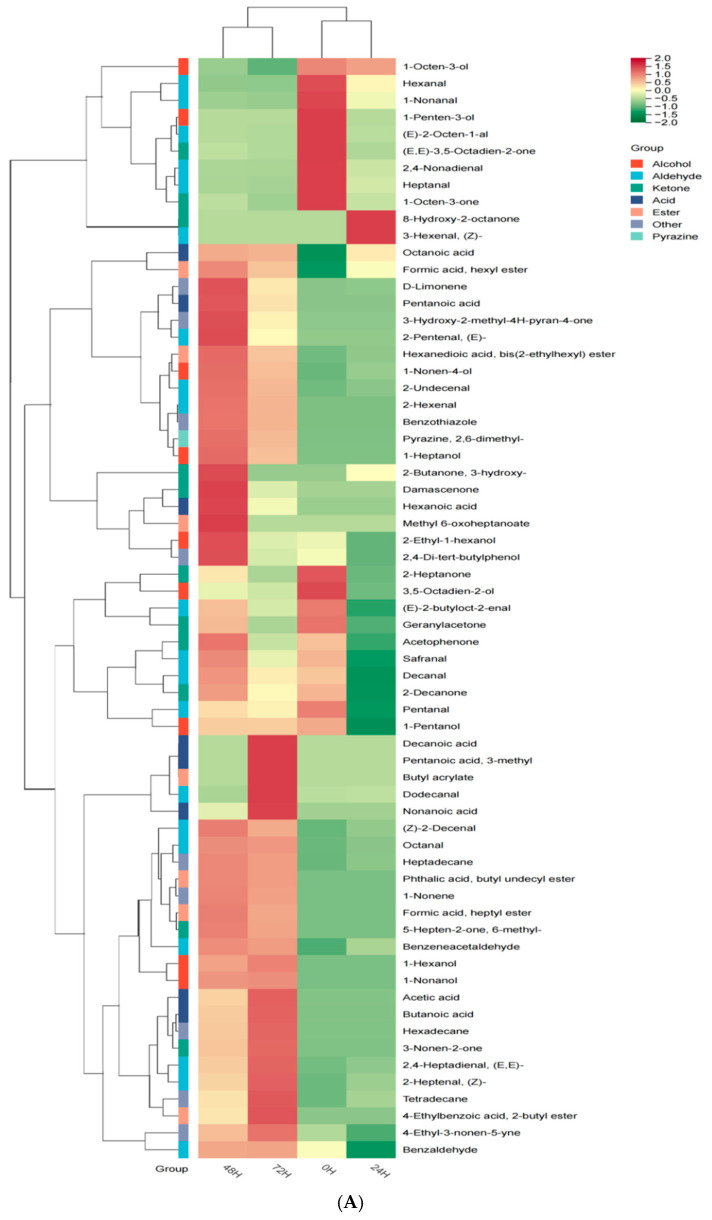

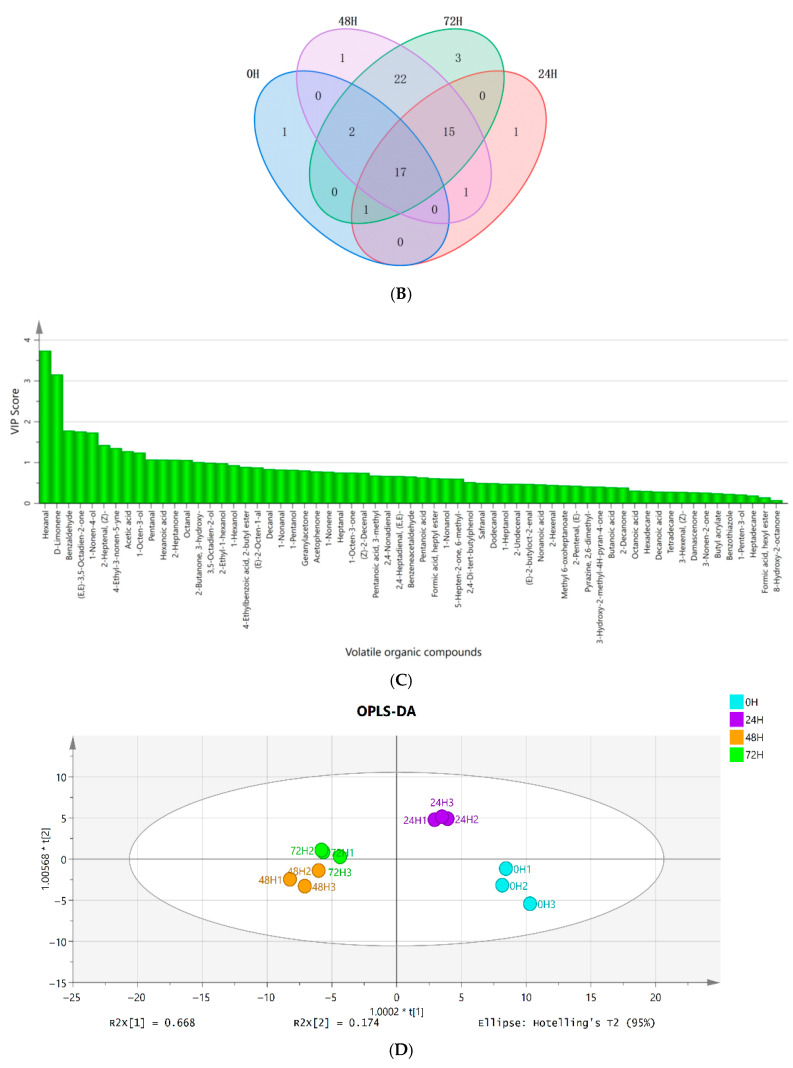

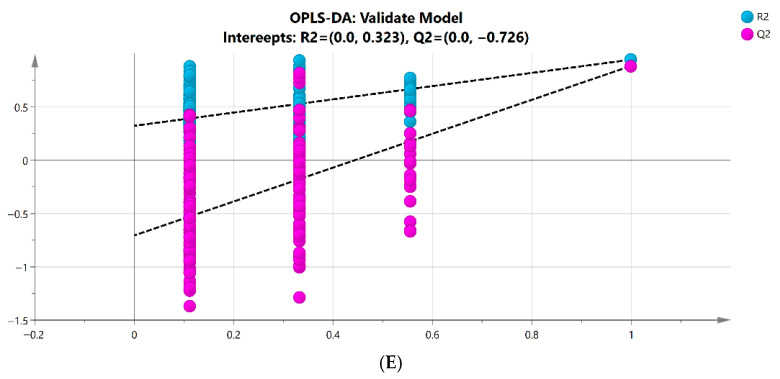
Research on volatile components of pea protein under different fermentation times hierarchical cluster analysis and heat map (**A**). Venn diagram (**B**). VIP score map of PLS-DA (**C**). Orthogonal partial least squares discriminant analysis (OPLS-DA) (**D**). Permutation test of OPLS-DA at 200-fold (R2: predictive power of the model, Q2: interpretability of the model) (**E**).

Among both unfermented and fermented pea proteins, aldehydes were the most abundant volatile components. In the unfermented pea protein, a total of 12 aldehyde compounds were detected. Previous studies have indicated that hexanal, nonanal, heptanal, and (E)-2-octenal are generated through the oxidation of proteins and lipids in legumes, contributing to the characteristic and undesirable fishy odor associated with these proteins [49]. Among them, hexanal combines with other flavor components (such as 1-octen-3-ol, 1-octen-3-one, etc.) and generates an unacceptable bean-like off-flavor at different concentrations. Hexanal has been predicted to be the primary contributor to the characteristic bean-like off-flavor of peas. OPLS-DA model analysis (Figure 5C) indicated that the VIP value of hexanal exceeded one, demonstrating its dominant contribution to the model. Further analysis revealed that in unfermented pea protein, the concentration of hexanal reached 57.16 mg/kg, representing the highest level among all detected volatile components. During the 72 h fermentation period, the concentration of hexanal steadily declined relative to its initial level, reaching a minimum of 5.09 mg/kg at the end of fermentation, representing a significant reduction of 92% compared to the unfermented PPI (*p* < 0.05). This significant decline might be explained related to the presence of alcohol dehydrogenase (ADH) in the metabolic process of lactic-acid bacteria [15]. Previous studies have reported that alcohol dehydrogenase (ADH) can catalyze the conversion of aldehydes to primary alcohols and ketones to secondary alcohols. Notably, hexanoic acid was undetectable during the initial 24 h of fermentation; however, as fermentation proceeded to 48 h, its concentration reached a maximum of 2.57 mg/kg, suggesting that a portion of hexanal may have been transformed into hexanoic acid [50]. Meanwhile, caproic acid can impart a certain degree of sweetness and refreshing flavor to food. Nonanal has unpleasant odors such as waxy and grassy scents [51]. After 72 h of bacterial fermentation, the content of nonanal decreased significantly from 2.21 mg/kg to 0.31 mg/kg (*p* < 0.05), contributing positively to the improvement of the PPI aroma. In addition, the levels of heptanal and E-2-octenal also declined markedly following fermentation (*p* < 0.05), with reductions of 93.05% and 65.8%, respectively. Benzaldehyde was found to be a typical flavor component in Kazakh cheese, with the aroma of bitter almonds and nuts. However, unlike other studies, it showed a significant increase after 48 h and 72 h of fermentation (*p* < 0.05), which might be due to the degradation of phenylalanine forming [52]. Six new aldehyde substances were detected after fermentation. Interestingly, in the fermented pea protein, phenylethanal was detected by SPME-GC/MS. Zhao, Chen et al. [9] also detected this component in their research. Phenylethanal can impart floral and honey-like pleasant scents. It can be derived from phenylalanine through the decarboxylation catalyzed by aromatic L-amino acids [53]. Studies have shown that their contents are relatively high in fermented sheep-liver paste [54].

The orthogonal partial least squares discriminant analysis indicated that there were significant differences in the flavor of pea protein obtained from four different fermentation periods. During the fermentation process, the types of compounds such as ketones, acids, and esters also experienced corresponding up-regulation. Based on the analysis results of the OPLS-DA model, VIP is a commonly used parameter for evaluating the contribution degree of variables. VIP > 1.0 can reflect the differences of the statistical model [55]. Following screening (Table 4), it was observed that among the alcohols, 1-octen-3-ol and 1-nonen-4-ol made the most substantial contributions to the model. Notably, 1-octen-3-ol is associated with a characteristic fishy odor in legumes [49]. After conducting experiments, it was found that the concentration of 1-octadecen-3-ol did not show significant changes after 24 h of fermentation. It merely decreased from 6.95 (mg/kg) to 6.54 (mg/kg) at the beginning. After 72 h of fermentation, the content of 1-octadecen-3-ol significantly decreased (*p* < 0.05), and the final concentration of 1-octadecen-3-ol was found to be 1.73 (mg/kg), effectively reducing the level of compounds causing the bean flavor in pea protein. Moreover, some new aroma compounds emerged during fermentation, including D-limonene. Through OPLS-DA model analysis, it was known that D-limonene contributed to the flavor of the fermented pea protein second only to hexanal. Its content reached the maximum value of 23.21 (mg/kg) after 48 h of fermentation, accounting for 22.18%. Studies have reported that D-limonene, identified as a potential flavor compound in yak beef jerky [56], is also abundantly present in essential oils and fragrances [57]. These changes may enhance the overall palatability of the fermented pea protein. During fermentation, the concentration of acetic acid increased markedly, reaching 3.02 mg/kg after 72 h. However, owing to its relatively high sensory threshold, the overall flavor profile of the pea-protein isolate remained largely unaffected.

Initially, no ester compounds were detected in the unfermented pea protein. Following fermentation, several esters—including adipic acid di-(2-ethylhexyl) ester, phthalic acid mono-nonadecyl ester, methyl formate, 4-ethylbenzoic acid ethyl ester, and methyl heptanoate—were generated, which could contribute desirable floral or fruity aromas to the protein [58]. Overall, the formation of ester compounds played an important role in masking undesirable flavors and enhancing the sensory attributes of fermented pea protein.

The main source of ketones during fermentation is produced through the oxidation of saturated β-fatty acids and the degradation of amino acids [59]. After 24 h and 48 h fermentation, 3-hydroxy-2-butanone was detected in the pea protein, which was similar to the result of Liu, Sun et al. [60], Moreover, 3-hydroxy-2-butanone could exhibit a rich buttery aroma.

In conclusion, bacterial fermentation of pea protein markedly reduced the levels of furfu ral (corncob-like odor), 1-octen-3-ol (earthy and mushroom-like odor), and nonanal (soybean-like odor) (*p* < 0.05). Moreover, compared to unfermented pea protein, fermentation generated new aroma compounds, which may more effectively mitigate or mask the characteristic off-flavors of pea protein.

**Table 4:** VIP value, concentration of volatile flavor substances and aroma characteristics.

Volatile Organic Compounds	Volatile Organic Compounds Content						
	Odor characteristic	RT	F-0H	F-24H	F-48H	F-72H	VIP
Hexanal	grassy	5.92	57.63 ± 8.06 ^a^	31.11 ± 1.56 ^b^	10.42 ± 2.85 ^c^	5.09 ± 0.95 ^c^	3.74
D-Limonene		16.14	—	0.32 ± 0.03 ^c^	23.21 ± 3.97 ^a^	10.51 ± 3.25 ^b^	3.15
Benzaldehyde	almond	13.16	5.09 ± 1.22 ^b^	1.34 ± 0.10 ^c^	7.13 ± 0.7 ^a^	7.20 ± 0.88 ^a^	1.78
(E,E)-3,5-Octadien-One	vegetable, hay	18.07	7.34 ± 5.70 ^a^	0.61 ± 0.58 ^c^	1.42 ± 0.41 ^b^	1.09 ± 0.21 ^b^	1.76
1-Nonen-4-ol		19.19	—	1.16 ± 0.29 ^c^	9.29 ± 1.48 ^a^	6.15 ± 0.55 ^b^	1.74
2-Heptenal-Z	almond, fruit	12.63	—	0.51 ± 0.04 ^c^	2.32 ± 0.32 ^b^	3.93 ± 0.79 ^a^	1.43
4-Ethyl-3-nonen-5-yne		16.56	1.30 ± 1.51 ^bc^	0.32 ± 0.03 ^c^	3.01 ± 1.17 ^a^	3.96 ± 0.82 ^a^	1.35
Acetic acid	acid	3.02	—	—	1.73 ± 0.23 ^b^	3.02 ± 0.06 ^a^	1.28
1-Octen-3-ol	mushroom, vegetable	14.16	6.94 ± 1.53 ^a^	6.54 ± 3.52 ^a^	3.44 ± 0.36 ^b^	1.73 ± 0.34 ^c^	1.24
Pentanal	bread, fruity	3.17	2.90 ± 1.42 ^a^	0.62 ± 0.04 ^b^	2.20 ± 0.47 ^a^	2.05 ± 0.30 ^a^	1.07
Hexanoic acid	meat broth	15.45	—	—	2.57 ± 0.43 ^a^	0.72 ± 0.31 ^b^	1.07
2-Heptanone	cheese, fruit	10.09	2.62 ± 1.32 ^a^	0.42 ± 0.01 ^b^	1.53 ± 0.43 ^ab^	0.72 ± 0.31 ^b^	1.06
Octanal	citrus, fat, green	15.11	—	0.27 ± 0.01 ^b^	2.89 ± 0.47 ^a^	2.77 ± 0.94 ^a^	1.06
2-Butanone,3-hydnxy-	butter, creamy	3.38	—	0.42 ± 0.03 ^ab^	1.24 ± 0.09 ^a^	—	1.01
3,5-Octadien-2-ol		16.83	2.33 ± 0.4 ^a^	0.17 ± 0.03 ^c^	0.84 ± 0.08 ^b^	0.69 ± 0.06 ^b^	0.99
2-Enthyl-1-hexanol		16.29	0.72 ± 0.08 ^b^	—	1.97 ± 0.35 ^a^	0.63 ± 0.10 ^b^	0.98

Note: Values are means ± SD. Different letters indicate that the effect of different fermentation time on the same compound was significantly different (*p* < 0.05).”—" Not detected. Odor descriptions were cited from www.flavornet.org and recent reports.

### 3.6. E-Tongue Analysis

**Figure 6:**
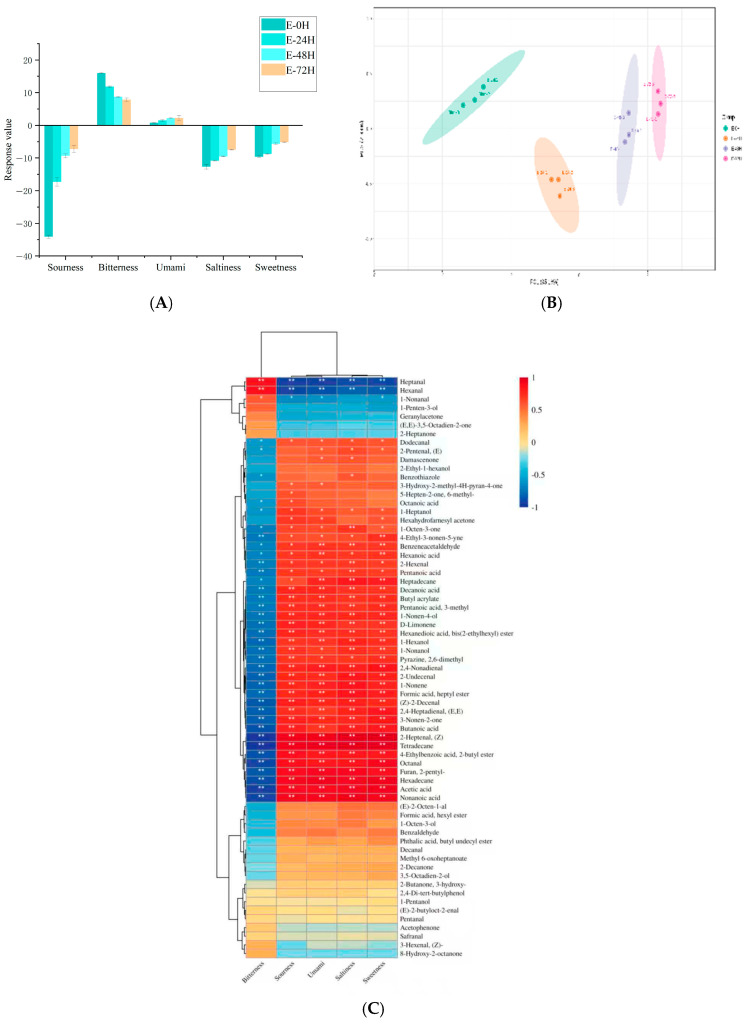

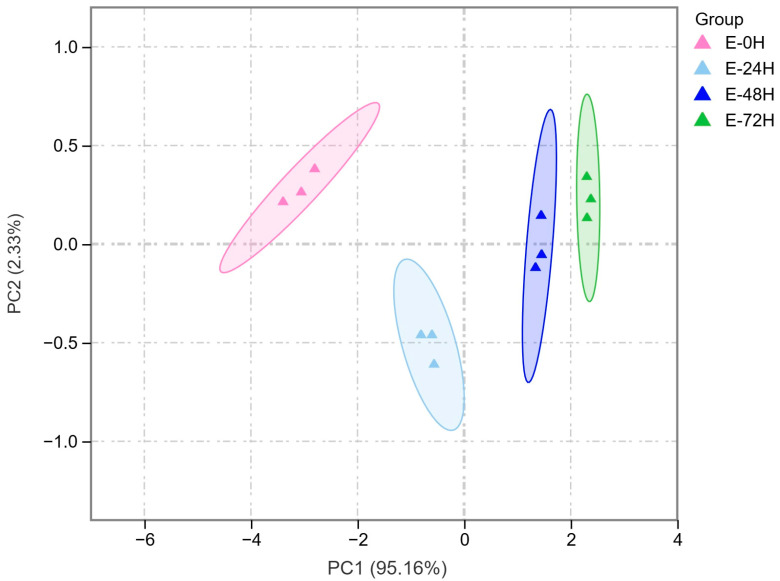

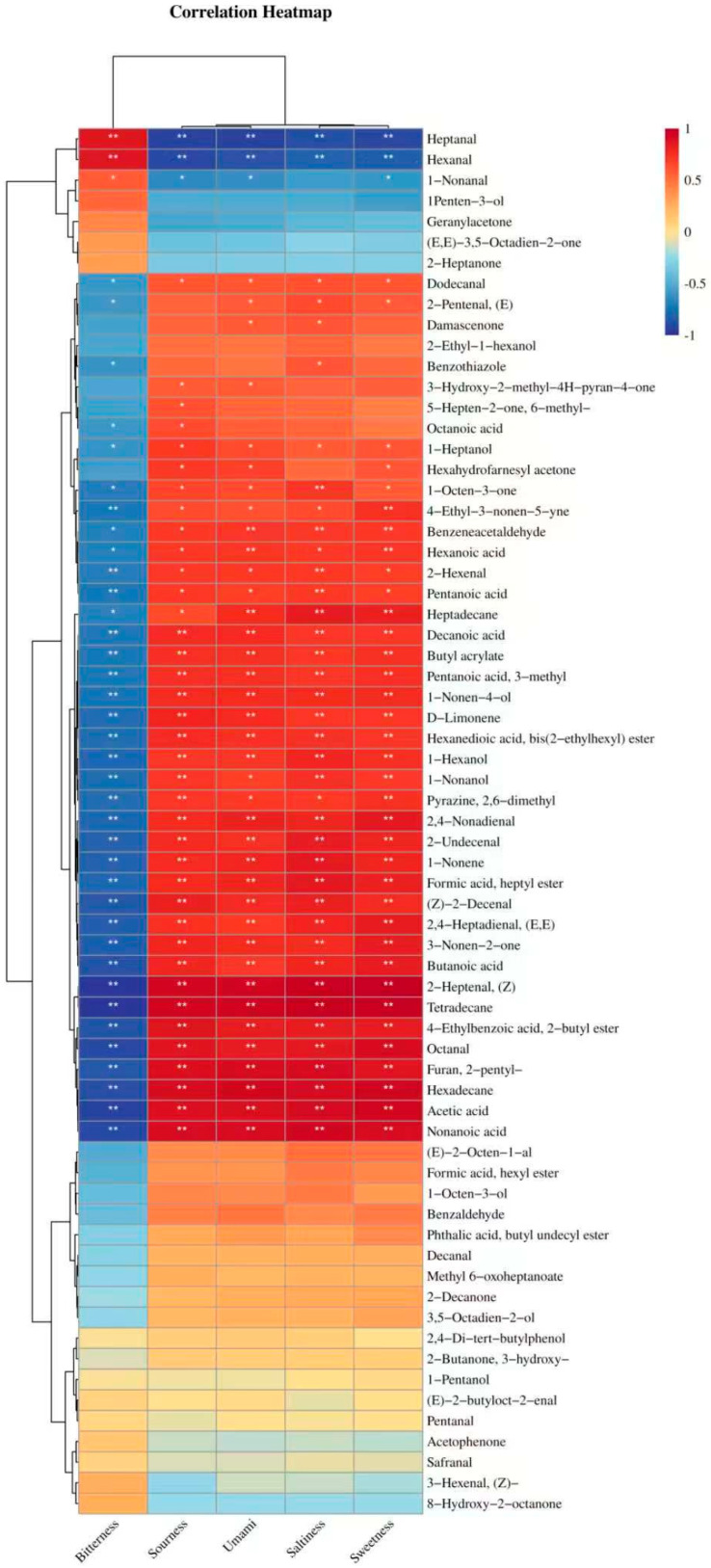
Electric-tongue response value plot for pea protein isolate (PPI) fermented with *Enterococcus faecalis* 07, E-0H,E-24H,E-48H,E-72H fermented 0 H, 24 H, 48 H, 72 H with *Enterococcus faecalis* 07 (**A**). Electric-tongue response value plot for pea protein isolate (PPI) fermented with Enterococcus faecalis07 PCA (**B**). Correlation network diagram between electronic nose and volatile flavor compounds. Red indicates a positive correlation, and blue indicates a negative correlation; the darker the color, the stronger the correlation. * indicates 0.01 < *p* ≤ 0.05; ** indicates 0.001 < *p* ≤ 0.01; a greater number of asterisks indicates higher significance, while the absence of asterisks indicates no significant correlation (**C**).

According to the results presented in Figure Figure 6A, significant differences in taste per-ception were observed in PPI fermented by cocci. Compared with unfermented PPI, the sample fermented with *Enterococcus faecalis* 07 exhibited notable changes, particularly in bitterness and umami. In terms of bitterness, a significant decrease was observed with prolonged fermentation time (*p* < 0.05), with the E-72H sample showing the lowest bitterness intensity (score: 7.85). The observed reduction in bitterness is likely attributable to the degradation of bitter-tasting amino acids, such as arginine (Arg) and histidine (His), during fermentation. Concurrently, as illustrated in Figure 6A, the umami response intensity progressively increased (*p* < 0.05), which may be associated with the accumulation of umami-contributing amino acids generated throughout the fermentation process.

Principal component analysis (PCA) enables the visualization of differences among samples and is specifically used to identify variations between them [61]. As shown in Figure 6B, PCA was performed on samples from different fermentation stages, with the two axes (PC1 and PC2) representing the overall taste characteristics of each fermented sample. The cumulative variance contribution rates of PC1 (95.16%) and PC2 (2.3%) reached 97.46%, indicating that the PCA plot captured the majority of the data variability, making PCA an effective tool for analyzing the taste profiles of the samples. Among them, the PPI that underwent 72 h fermentation was the farthest from the non-fermented group. This indicates that as the fermentation time increased, it had a significant impact on the taste of the PPI.

### 3.7. Correlation Analysis Between E-Tongue and Volatile Compounds

As illustrated in Figure 6A, fermentation of PPI by *Enterococcus faecalis* 07 induced notable changes in both bitterness and umami perceptions. Bitterness exhibited a strong positive correlation with only two volatile compounds, heptanal and hexanal (*p* < 0.05, |r| > 0.8), while demonstrating negative correlations with 29 other volatile compounds. In contrast, umami was negatively correlated with three compounds (heptanal, hexanal, and 1-nonanal; *p* < 0.05, |r| > 0.8), but positively correlated with 26 volatiles (Figure 6C). The decreased levels of the three umami-associated negatively correlated volatiles suggest that prolonged fermentation reduced their concentrations through coccal metabolism, thereby enhancing umami perception. Sourness was positively correlated with the majority of volatile compounds, indicating that coccal fermentation enhanced acidity and altered the volatile profile, thereby influencing sourness perception. Similarly, saltiness and sweetness exhibited comparable patterns, demonstrating the impact of volatile compounds on these sensory attributes.

### 3.8. Sensory Evaluation

**Figure 7:**
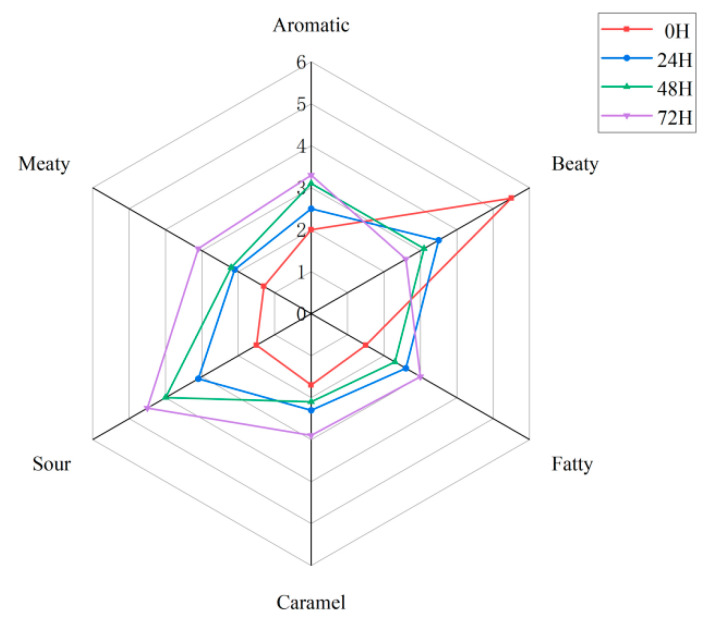
Sensory evaluation of pea protein. The red line represents the fermentation at 0 H, the blue line represents the fermentation at 24 H, the green line represents the fermentation at 48 H, and the purple line represents the fermentation at 72 H.

Figure 7 presents the odor profiles of pea-protein samples following fermentation. Overall, fermentation not only diminished the undesirable beany odor but also enhanced favorable sensory attributes, including aromatic, meaty, and caramel-like notes, as fermentation time increased. The observed increase in sourness is likely attributable to the accumulation of organic acids produced during fermentation. Furthermore, elevated levels of esters, alcohols, and ketones appear to have contributed to the enhanced aromatic perception. Notably, the sample fermented for 72 h exhibited the most acceptable overall odor profile, suggesting that prolonged fermentation can promote the development of more consumer-appealing flavor characteristics.

## 4. Conclusions

This study investigated the fermentation process of *Enterococcus faecalis* 07 using pea protein as the substrate. During the fermentation process, the amino acid composition, physicochemical properties, and flavor compounds of pea protein isolate changed, which enhanced the water-holding and oil-holding properties of PPI. The reduction of the concentrations of undesirable flavors such as hexanal (herbaceous flavor), 1-octen-3-ol (mushroom flavor), nonanal (soybean-like flavor), and E-2-octen-1-ol (herbaceous flavor) was of great significance for improving the flavor of pea protein. These results indicate that fermentation with *Enterococcus faecalis* 07 can effectively enhance the sensory and physicochemical properties of plant proteins, offering a promising strategy for their application in high-quality, palatable food products. Overall, this study demonstrates the potential of this strain in improving PPI flavor; however, the underlying metabolic pathways responsible for flavor formation, as well as the effects of protein conformational changes on the binding of beany flavor compounds, require further investigation in future research. This study not only provides a scientific basis for the application of *E. faecalis* 07 in plant-based foods but also offers important insights for the efficient utilization of pea protein in food processing.

## Figures and Tables

**Table 1:** The changes in organic acid content of pea protein during the fermentation process.

Organic Acid	Organic Acid Content (mg/mL)			
	F-0H	F-24H	F-48H	F-72H
Lactic acid	ND	3.53 ± 0.12 ^c^	4.21 ± 0.14 ^b^	4.55 ± 0.46 ^a^
Formic acid	ND	1.38 ± 0.16 ^b^	1.81 ± 0.05 ^a^	1.54 ± 0.33 ^b^
Tartaric acid	0.05 ± 0.01 ^d^	4.51 ± 0.07 ^b^	4.75 ± 0.01 ^a^	4.13 ± 0.07 ^c^
Malic acid	0.09 ± 0.01 ^c^	0.47 ± 0.01 ^a^	0.19 ± 0.07 ^b^	0.12 ± 0.05 ^b^
Citric acid	0.09 ± 0.02 ^b^	0.15 ± 0.03 ^a^	0.19 ± 0.03 ^a^	0.14 ± 0.06 ^a^
Succinic acid	0.09 ± 0.01 ^d^	0.19 ± 0.06 ^b^	0.14 ± 0.03 ^c^	0.24 ± 0.01 ^a^
Fumaric acid	ND	ND	0.06 ± 0.02 ^a^	0.04 ± 0.01 ^a^

Note: ND indicates undetected. The values are the average ± standard deviation. Different letters within the same column represent significant differences among samples (*p* < 0.05). F-0H represents fermentation 0 h, F-24H represents fermentation for 24 h, F-48H represents fermentation for 48 h, and F-72H represents fermentation for 72 h.

**Table 2:** Changes in the nucleotide content of pea protein during the fermentation process.

5′-Nucleotide	Nucleotide Content (mg/100 g)			
	F-0H	F-24H	F-48H	F-72H
5′-AMP	7.10 ± 0.19 ^c^	7.55 ± 0.24 ^bc^	8.81 ± 0.92 ^ab^	9.24 ± 0.95 ^a^
5′-GMP	6.14 ± 0.06 ^b^	6.09 ± 0.03 ^b^	6.31 ± 0.1 ^b^	7.62 ± 0.58 ^a^
5′-CMP	1.57 ± 0.2 ^c^	1.66 ± 0.03 ^bc^	1.87 ± 0.06 ^ab^	1.94 ± 0.1 ^a^

Note: The values are the average ± standard deviation. Different letters within the same column indicate significant differences among samples (*p* < 0.05).

**Table 3:** Variations in the level of free amino acids before and after fermentation.

Free Amino Acid	Free Amino-Acid Content (mg/100 g)			
	F-0H	F-24H	F-48H	F-72H
Asp	2.36 ± 0.06 ^b^	9.20 ± 0.71 ^a^	9.37 ± 0.58 ^a^	9.52 ± 0.59 ^a^
Thr	1.35 ± 0.02 ^c^	2.64 ± 0.12 ^b^	2.72 ± 0.25 ^ab^	2.97 ± 0.11 ^a^
Ser	0.9 ± 0.05 ^a^	1.05 ± 0.1 ^a^	1.09 ± 0.11 ^a^	1.05 ± 0.11 ^b^
Glu	21.25 ± 1.47 ^a^	17.13 ± 0.29 ^b^	17.59 ± 0.02 ^b^	16.49 ± 0.45 ^b^
Gly	19.57 ± 0.78 ^b^	25.63 ± 0.99 ^a^	24.52 ± 0.07 ^a^	25.62 ± 0.72 ^a^
Ala	4.92 ± 0.10 ^b^	5.65 ± 0.49 ^a^	5.93 ± 0.55 ^a^	5.93 ± 0.41 ^a^
Cys	0.63 ± 0.01 ^b^	1.42 ± 0.36 ^a^	1.39 ± 0.26 ^a^	1.34 ± 0.01 ^a^
Val	9.73 ± 0.10 ^c^	12.37 ± 0.29 ^b^	12.92 ± 0.18 ^a^	12.41 ± 0.25 ^b^
Met	0.54 ± 0.02 ^b^	0.98 ± 0.25 ^a^	0.61 ± 0.08 ^b^	0.57 ± 0.03 ^b^
Ile	7.09 ± 0.01	7.24 ± 0.26	6.55 ± 0.63	6.50 ± 0.60
.Leu	16.68 ± 0.36 ^c^	18.23 ± 0.77 ^a^	17.64 ± 0.55 ^b^	18.48 ± 1.42 ^a^
Tyr	7.52 ± 0.18 ^a^	7.01 ± 0.21 ^b^	6.93 ± 0.17 ^b^	6.72 ± 0.25 ^b^
Phe	6.49 ± 0.18 ^c^	8.81 ± 0.25 ^b^	9.57 ± 0.41 ^a^	9.66 ± 0.42 ^a^
Lys	7.75 ± 0.26 ^c^	8.52 ± 0.18 ^b^	9.23 ± 0.37 ^a^	9.54 ± 0.39 ^a^
His	4.33 ± 0.17 ^c^	5.82 ± 0.74 ^ab^	4.92 ± 0.36 ^bc^	6.80 ± 0.98 ^a^
Arg	20.94 ± 0.09 ^a^	10.94 ± 0.40 ^b^	10.81 ± 0.46 ^b^	10.74 ± 0.25 ^b^
Umami amino acids	24.51 ± 1.42 ^b^	26.33 ± 0.82 ^ab^	27.26 ± 0.58 ^a^	25.51 ± 0.90 ^ab^
Sweet amino acids	26.7 ± 0.74 ^b^	34.92 ± 1.49 ^a^	34.19 ± 0.67 ^a^	35.57 ± 0.36 ^a^
Bitter amino acids	73.32 ± 0.83 ^a^	70.19 ± 0.68 ^b^	68.87 ± 0.63 ^b^	70.93 ± 1.78 ^b^

Note: The values are the average ± standard deviation. Different letters within the same column indicate significant differences among samples (*p* < 0.05).

## Data Availability

The original contributions presented in this study are included in the article. Further inquiries can be directed to the corresponding author.
